# Linear and Non-Linear Associations of Gonorrhea Diagnosis Rates with Social Determinants of Health

**DOI:** 10.3390/ijerph9093149

**Published:** 2012-09-03

**Authors:** Ramal Moonesinghe, Eleanor Fleming, Benedict I. Truman, Hazel D. Dean

**Affiliations:** 1 Centers for Disease Control and Prevention, National Center for HIV/AIDS, Viral Hepatitis, STD, and TB Prevention, Atlanta, GA 30333, USA; Email: efleming@cdc.gov (E.F.); btruman@cdc.gov (B.I.T.); hdean@cdc.gov (H.D.D.); 2 Centers for Disease Control and Prevention, Division of Applied Sciences, Epidemic Intelligence Service, Scientific Education and Professional Development Program Office, Atlanta, GA 30333, USA

**Keywords:** social determinants of health, gonorrhea diagnosis rates, product moment correlation, maximal information coefficient

## Abstract

Identifying how social determinants of health (SDH) influence the burden of disease in communities and populations is critically important to determine how to target public health interventions and move toward health equity. A holistic approach to disease prevention involves understanding the combined effects of individual, social, health system, and environmental determinants on geographic area-based disease burden. Using 2006–2008 gonorrhea surveillance data from the National Notifiable Sexually Transmitted Disease Surveillance and SDH variables from the American Community Survey, we calculated the diagnosis rate for each geographic area and analyzed the associations between those rates and the SDH and demographic variables. The estimated product moment correlation (PMC) between gonorrhea rate and SDH variables ranged from 0.11 to 0.83. Proportions of the population that were black, of minority race/ethnicity, and unmarried, were each strongly correlated with gonorrhea diagnosis rates. The population density, female proportion, and proportion below the poverty level were moderately correlated with gonorrhea diagnosis rate. To better understand relationships among SDH, demographic variables, and gonorrhea diagnosis rates, more geographic area-based estimates of additional variables are required. With the availability of more SDH variables and methods that distinguish linear from non-linear associations, geographic area-based analysis of disease incidence and SDH can add value to public health prevention and control programs.

## 1. Introduction

Public health practitioners, researchers, and policy makers are examining the social determinants of health (SDH) to better understand the health of communities and to improve population health through targeted interventions. Knowing that a particular group bears excess burden of a disease is important, but knowing how the SDH influence that excess burden of disease is perhaps more important and useful in achieving health equity. The Centers for Disease Control and Prevention’s (CDC’s) National Center for HIV/AIDS, Viral Hepatitis, Sexually Transmitted Disease (STD), and Tuberculosis (TB) Prevention (NCHHSTP) uses a holistic, science-based approach that is modeled on the World Health Organization’s framework on SDH to promote health equity and improve the health of communities [1]. A holistic approach to disease prevention involves addressing the combined effects of individual, social, health system, and environmental determinants through combined efforts of all societal sectors, such as health, education, justice, environment, and labor [2]. In addition, holistic intersectoral work involves the use of diverse sources and kinds of data, including disease surveillance, legal, land use, marketing, workforce, education, and financial data [2]. Practitioners, policy makers, and researchers using surveillance, housing and labor, gender equity, and policy data are able to measure and quantify the effects of SDH at neighborhood, city, county, state, and national levels [3,4,5,6,7,8].

Gonorrhea is the second most common notifiable disease in the United States. In 2009, 301,174 cases of gonorrhea were reported—the lowest number ever recorded. Although gonorrhea rates had been declining since the mid 1970s, group-specific rates by sex, age, and race/ethnicity increased between 2009 and 2010. Reported gonorrhea diagnosis rate is highest among young women aged 15–24 years and race/ethnic disparities in gonorrhea diagnosis rates are among the highest for any disease with non-Hispanic black persons bearing a disproportionate burden of disease in the United States [9]. Gonorrhea diagnosis rates have been highest among non-Hispanic black persons for the past decade [9]. In 2010, the rate among non-Hispanic blacks (435.5 cases per 100,000 population) was 18.7 times the rate of non-Hispanic whites (23.1 per 100,000 population) [9]. In 2010, 69% of all reported cases of gonorrhea occurred among non-Hispanic black persons. Individual risk factors, such as age, number of sex partners, past history of STD, illicit drug use, and new or multiple sex partners are important; however, social, health system and other environmental factors beyond the control of individuals also influence this disparity [10,11,12,13].

Understanding gonorrhea in the context of SDH also is important in the prevention of disease complications and antibiotic resistance. Among women, gonorrhea causes a spectrum of upper genital tract infections that can lead to infertility, ectopic pregnancy, and chronic pelvic pain—common consequences of pelvic inflammatory disease. Gonorrhea also increases the risk of HIV transmission. Growing cephalosporin antibiotic resistance and fewer alternative treatment options increase the importance of decreasing gonorrhea morbidity, perhaps by modifying those social, health system, and other environmental factors that are increasing population risk [14]. Understanding the behavioral and social determinants is central to reducing the spread of STDs, and with area-based SDH measures, we can better understand gonorrhea disparities. 

Song *et al*. used 2006–2008 American Community Survey (ACS) and National HIV Surveillance System data to measure the impact of socioeconomic factors on AIDS diagnosis rates [5]. With correlation and partial correlation coefficients, the analysis identified HIV risk factors and showed the added effects of interactions between SDH variables. Song *et al.* illustrated a method for using national surveillance data and national area-based demographic data to better describe the potential impact of SDH on the geographic distribution of disease morbidity. They showed that AIDS diagnosis rates were highly correlated with race/ethnicity, population density, and marital status. 

In this article, which is focused on statistical methods in order to replicate the methods described by Song *et al*. and to extend their applicability, we conducted exploratory statistical analyses to identify associations among gonorrhea diagnosis rates and SDH variables. We discovered associations between pairs of variables using coefficients of correlations and also investigated the association between gonorrhea diagnosis rate and a SDH variable while controlling for another SDH variable using partial correlations. Since the method used to calculate correlations assume linear relationships, we examined non-linear associations among SDH and gonorrhea diagnosis rate. Lastly, we evaluated how well non-linear associations describe the relationships between SDH and gonorrhea diagnosis rates, which may be useful in public health research and practice.

## 2. Methods

To identify the relationship between SDH variables and AIDS diagnosis rates, Song *et al*. analyzed ACS population data by geographic area of residence [5]. The ACS is a nationwide survey that collects same information on people and housing status that was collected on the long form questionnaire used in past decennial censuses. The ACS also collects information on socioeconomic variables and the sample size of ACS is about 1% of the total population each year. Due to confidentiality requirements, the available Gonorrhea surveillance data were aggregated by geographic area for linkage with available data in the ACS. Because the public-use micro-data area (PUMA) in the ACS may be small in terms of square miles in population-dense areas, the authors defined an area, called county-PUMA overlap area (CPOA), as either a county or a PUMA (whichever was larger) so that the area could be identified in both the ACS data and the case surveillance data. In the United States, a county is a geographic subdivision of a state with some governmental authority. We linked CDC, NCHHSTP STD Surveillance System data with the SDH and demographic variables from ACS used by Song *et al.* to study the associations between the estimated diagnosis rate of gonorrhea and the following SDH variables: population density (dens); proportion female (p_female); proportion aged 30 or less years (p_young); proportion Hispanic (p_hisp); proportion non-Hispanic black (p_black); proportion of minority race/ethnicity (p_xwhite); proportion not currently married (p_single); proportion below the FPL (p_pov); proportion with less than a high school education (p_hsch); proportion unemployed (p_unemp); proportion moved in the past 12 months (p_moved); and proportion foreign-born (p_foreign).

Gonorrhea diagnosis rates were computed from data collected in the National Notifiable STD Surveillance System. STD control programs and health departments in all 50 states, the District of Columbia, selected cities, Guam, Puerto Rico, and the Virgin Islands submit STD morbidity data in hard copy and electronically (via the National Electronic Telecommunications System for Surveillance) to the Division of STD Prevention, NCHHSTP, CDC. Diagnosis rate for each CPOA was calculated using data on people with a diagnosis of gonorrhea (age 15 years and above) reported to CDC between 2006 and 2008. If the number of cases reported for each CPOA within this period was less than 30, we included cases reported to CDC between 2003 and 2005. Based on this criterion, 30 of the 949 CPOAs contained data from the 2003–2005 period. Rates per 100,000 person-years were calculated for each CPOA. 

We estimated Pearson product moment correlations (PMCs) between gonorrhea diagnosis rate and SDH and demographic variables. Because PMC measures linear relationships, we examined the relationships between gonorrhea diagnosis rate and other variables using scatter plots to detect non-linear patterns. To make these relationships more linear, we log-transformed the following variables: gonorrhea diagnosis rate (log_rate); population density (log_dens); proportions of foreign-born (q_foreign); Hispanic (q_hisp); non-Hispanic black (q_black); and all racial/ethnic minority groups (q_xwhite).

We also estimated the first order partial correlations between gonorrhea diagnosis rate and each SDH and demographic variable. The partial correlation between two variables, X and Y, given a set of n controlling variables **Z** = (Z_1_, Z_2_,…, Zn), is the correlation between the residuals resulting from the multiple linear regression of X with **Z** and of Y with **Z**, respectively. The order of correlation, n, is the number of controlled variables. Partial correlation measures the strength of a linear relationship between two variables while controlling for a third or more than one other variables. When there is not much difference between partial correlation and PMC, one could conclude that there is no or minimal effect of the control variables **Z **on the association of variables X and Y. 

PMC captures only the strength of linear association and its usefulness is greatly reduced when associations are non-linear [15]. When variables are transformed to estimate PMC, it measures the strength of the linear association between transformed variables. What has long been needed is a measure that directly quantifies associations between two variables whether or not they are linear [15]. Recently, Reshef *et al*. developed a novel, statistical approach to uncover non-linear associations in large data sets [16]. They presented a measure of dependence for two-variable relationships—the maximal information coefficient (MIC). MIC captures a wide range of associations—both functional and otherwise—and for functional relationships, provides a score that roughly equals the coefficient of determination (R^2^) of the data relative to the regression function [16]. The coefficient of determination measures how well the regression curve approximates the observed data points. The algorithm proposed by Reshef *et al.* assigns similar MIC scores to “equally noisy relationships of different types” [15]. The algorithm considers plots of every pair of variables in a dataset and then overlays each graph with a series of denser and denser grids and identifies the grid cells that contain data points. Using information theory, the algorithm assesses how orderly the patterns produced by the data-containing cells are and identify the most orderly pattern [17].

In a comparison of PMCs and MICs for noiseless functional relationships of cubic, exponential, sinusoidal (Fourier frequency), and parabolic forms, the PMCs ranged from −0.09 to 0.7, but the MICs were 1.0 for all the relationships [15]. When the functional relationship is linear, both the MIC and the PMC were 1 as expected. The authors provide a downloadable Java program, Maximal Information-based Nonparametric Exploration (MINE) application, to compute MICs for large datasets at the MINE website [18]. 

We used the MINE application to estimate MICs for the gonorrhea diagnosis rate and SDH variables. In addition to MIC, we also calculated a measure of non-linearity, MIC-ρ^2^, where ρ is the PMC [15]. The statistic MIC-ρ^2^ is near 0 for linear relationships and large for non-linear relationships with high values of MIC. Reshef *et al.* have pre-computed the p-values of various MIC scores at different sample sizes and made them available at the MINE Web site [18].

We also presented the strength of non-linear associations among SDH and demographic variables and gonorrhea diagnosis rate on a two-dimensional plane. Each variable is represented by a node in the graph and the relative strength of the association between two nodes is represented by the thickness of the line connecting the two nodes. We used the “qgraph” package in R software to create this graph [19].

## 3. Results

Based on the 2000 U.S. census data in the ACS, Song *et al.* defined 949 CPOAs in the 50 states and the District of Columbia. The diagnosis rate for gonorrhea for 2006–2008 was 117 per 100,000 person-years. The number of diagnosed cases within each CPOA ranged from 16 to 28,170 during this period. For the 30 CPOAs that had less than 30 gonorrhea cases, we calculated gonorrhea diagnosis rates for 2003–2008. Inclusion of the gonorrhea cases from 2003–2005, had minimal impact on the 2006–2008 gonorrhea diagnosis rates for these 30 CPOAs. The gonorrhea diagnosis rates in the 949 CPOAs varied from 3 to 684 cases per 100,000 person-years with a standard deviation of 103. To study the factors associated with such a large variation in the diagnosis rate for gonorrhea across CPOAs, we estimated the PMCs between diagnosis rates and each of the 12 demographic and SDH variables. 

Table 1 gives the estimated PMCs between gonorrhea diagnosis rate and socioeconomic and demographic variables (or their log transformations), and partial correlations between SDH variable X and the gonorrhea diagnosis rate, adjusted for SDH variable Y. The estimated PMCs between gonorrhea rate and SDH variables ranged from 0.11 to 0.83 and all the PMCs were significantly different from zero (*P* < 0.01). Proportions of the CPOA population that were black, of minority race/ethnicity, and unmarried were each strongly correlated with gonorrhea diagnosis rate with PMCs of 0.83, 0.65, and 0.61, respectively.

The partial correlation between gonorrhea diagnosis rate and the proportion of the CPOA population who are non-Hispanic black remains mostly strong (around 0.83) after controlling for all SDH variables individually. The exception is that the PMC is smaller (0.73) when controlling for proportion of CPOA population who are of any minority race/ethnicity or for proportion of CPOA population who are not currently married. This is to be expected since each of these variables is highly correlated with the gonorrhea diagnosis rate (around 0.63) and with the proportion of the CPOA population who are non-Hispanic black (>0.53) [5]. That is, each of these variables partially explains the original correlation between gonorrhea diagnosis rate and the proportion of the CPOA population who are non-Hispanic black. 

**Table 1:** Correlations between the gonorrhea diagnosis rate and both SDH variables and demographic variables, and partial correlations between variable X and gonorrhea diagnosis rate adjusted with variable Y, based on data at the CPOA level: National Notifiable STD Surveillance and American Community Survey data, 2006–2008.

			Y											
			F1	F2	F3	F4	F5	F6	F7	F8	F9	F10	F11	F12
X		log_rate	0.33	0.32	0.25	0.11	0.83	0.65	0.61	0.32	0.26	0.29	0.26	0.11
log_dens	(F1)	0.33		0.25	0.33	0.32	–0.10	0.21	0.18	0.50	0.36	0.32	0.35	0.32
p_female	(F2)	0.32	0.23		0.38	0.35	0.15	0.38	0.27	0.34	0.34	0.30	0.39	0.34
p_young	(F3)	0.25	0.25	0.32		0.22	0.22	–0.03	–0.05	0.17	0.14	0.24	0.12	0.22
q_hisp	(F4)	0.11	0.06	0.20	0.04		0.14	–0.55	0.02	0.15	–0.00	0.14	0.05	0.04
q_black	(F5)	0.83	0.81	0.81	0.83	0.83		0.72	0.75	0.82	0.82	0.82	0.84	0.83
q_xwhite	(F6)	0.65	0.62	0.67	0.62	0.77	0.31		0.44	0.63	0.62	0.64	0.63	0.74
p_single	(F7)	0.61	0.56	0.59	0.58	0.60	0.35	0.34		0.55	0.58	0.56	0.57	0.60
p_pov	(F8)	0.32	0.49	0.34	0.27	0.34	0.26	0.25	0.02		0.26	0.24	0.27	0.37
p_hsch	(F9)	0.26	0.30	0.28	0.17	0.24	0.21	–0.09	0.12	0.17		0.24	0.25	0.25
p_unemp	(F10)	0.29	0.28	0.27	0.28	0.30	0.15	0.23	0.04	0.20	0.27		0.29	0.30
p_moved	(F11)	0.26	0.28	0.34	0.15	0.24	0.31	0.11	0.08	0.19	0.25	0.25		0.24
q_foreign	(F12)	0.11	–0.06	0.16	0.05	0.03	–0.01	–0.48	–0.06	0.22	0.05	0.14	0.05	

log_rate = log(gonorrhea diagnosis rate); log_dens = log(population density) (F1); p_female = proportion female (F2); p_young = proportion aged ≤30 years (F3); q_hisp = log(proportion Hispanic) (F4); q_black = log(proportion non-Hispanic black); q_xwhite = log(proportion of minority race/ethnicity) (F6); p_single = proportion not currently married (F7); p_pov = proportion below the federal poverty level (F8); p_hsch = proportion with less than a high school education (F7); p_unemp = proportion unemployed (F10); p_moved = proportion moved in the past 12 months (F11); q_foreign = log(proportion foreign-born) (F12).

Similarly, the partial correlation between gonorrhea rate and the proportion of the CPOA population who are minority race/ ethnicity remains high (>0.62) except when controlling for proportion of the CPOA population who are non-Hispanic black or proportion of the CPOA population who are unmarried people (<0.44). There is a slight increase in the partial correlation when controlling for proportion of the CPOA population who are Hispanic or proportion of the CPOA population who are foreign-born, indicating that each of these two variables suppresses the correlation between gonorrhea rate and the proportion of the CPOA population who are minority race/ethnicity. The partial correlations between gonorrhea diagnosis rate and proportion of the CPOA population who are not currently married (around 0.6) only declined when controlled for proportion of the CPOA population who are non-Hispanic black or proportion of the CPOA population who are minority race/ethnicity (around 0.34).

The correlation between the gonorrhea diagnosis rate and the population density is 0.33, but the partial correlation when adjusted for the proportion of the CPOA population whose family incomes are below the federal poverty level, increases to 0.5. In other words, for the CPOAs with the same proportion of population below the federal poverty level, the correlation between the gonorrhea diagnosis rate and the population density is 0.5. On the other hand, when adjusted for proportion of the CPOA population who are non-Hispanic black, the partial correlation between the gonorrhea diagnosis rate and the population density declines to –0.10. Similarly, the correlation between gonorrhea diagnosis rate and proportion of the CPOA population whose family incomes are below the federal poverty level is 0.32, but when adjusted for population density, the partial correlation increases to 0.49. When this correlation is controlled for proportion of the CPOA population who are not currently married, the partial correlation is not significantly different from 0. Because the correlation of the proportion of CPOA population who are not currently married with proportion who live below the federal poverty level and the gonorrhea diagnosis rate are 0.51 and 0.61, respectively, the removal of the effects of proportion who are not currently married leads to the correlation between the gonorrhea diagnosis rate and proportion living below the federal poverty level collapsing to nearly zero. The correlation between the gonorrhea diagnosis rate and the proportion of the CPOA population who are foreign-born is only 0.11, but controlling for proportion of the CPOA population who are minority race/ethnicity, the partial correlation increases (absolutely) to –0.48. 

PMCs and partial correlations assume linear relationships. Generally, this assumption is not valid, as linear relationships rarely exist in areas such as social sciences. In our calculation of PMCs, we used log transformations for some variables so that the transformed variables have linear relationships. To study the non-linearity of these relationships, we estimated the non-linear associations using MICs.

**Figure 1:**
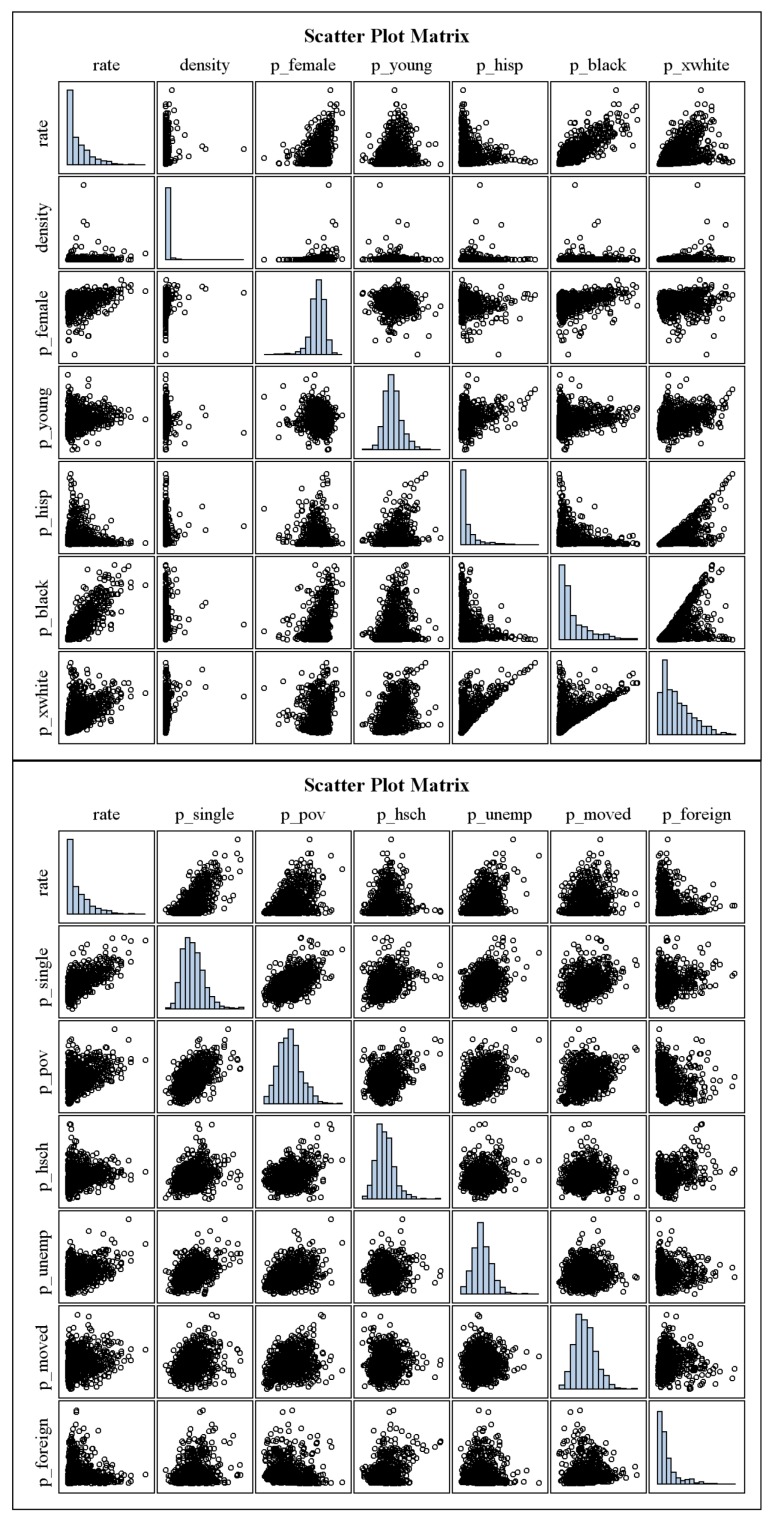
Histograms and scatter plots of the gonorrhea diagnosis rates and both SDH and demographic variables using the original variables (without log transformation): National Notifiable STD Surveillance and American Community Survey data, 2006–2008.

Figure 1 and Figure A1 in the Appendix show the histograms and scatter plots of the gonorrhea diagnosis rates and SDH and demographic variables using the original variables (without log transformation). The MIC, non-linearity measure, and PMC for each pair of variables are given in Table 2. The scatter plot for gonorrhea diagnosis rate and the proportion of the CPOA population who are non-Hispanic black (p_black) indicates that the relationship is linear. The PMC (rate, p_black) = 0.84 and MIC (rate, p_black) = 0.64. The difference between MIC and the coefficient of determination (0.84^2^ = 0.71) is the non-linearity measure (–0.07) that indicates the relationship between these two variables is almost linear. Because MIC measures the non-linear associations, the estimated MIC between two variables remains the same, regardless of whether the variables are transformed.

**Table 2:** Maximal Information Coefficient (MIC) Strength, non-linearity, and correlation between the gonorrhea diagnosis rate (GRDR) and SDH variables and demographic variables: National Notifiable STD Surveillance and American Community Survey data, 2006–2008.

X		GRDR	Y											
			F1	F2	F3	F4	F5	F6	F7	F8	F9	F10	F11	F12
MIC (Strength)														
Non-linearity														
Correlation														
dens	(F1)	0.21												
		0.19												
		0.11												
p_female	(F2)	0.21	0.18											
		0.08	0.16											
		0.37	0.14											
p_young	(F3)	0.20	0.14	0.15										
		0.14	0.14	0.12										
		0.23	–0.03	–0.17										
p_hisp	(F4)	0.16	0.19	0.17	0.19									
		0.16	0.17	0.14	0.07									
		–0.07	0.13	–0.18	0.35									
p_black	(F5)	0.63	0.29	0.22	0.19	0.17								
		–0.07	0.27	0.10	0.16	0.16								
		0.84	0.13	0.35	0.18	–0.12								
p_xwhite	(F6)	0.43	0.24	0.18	0.25	0.38	0.52							
		0.14	0.18	0.17	0.08	–0.04	0.17							
		0.54	0.23	0.07	0.41	0.65	0.60							
p_single	(F7)	0.37	0.17	0.17	0.28	0.18	0.31	0.39						
		–0.08	0.08	0.14	0.06	0.12	–0.09	0.02						
		0.67	0.29	0.18	0.46	0.23	0.64	0.61						
p_pov	(F8)	0.21	0.27	0.19	0.17	0.19	0.20	0.17	0.25					
		0.05	0.27	0.19	0.10	0.17	0.08	0.07	–0.01					
		0.40	–0.01	–0.01	0.28	0.13	0.35	0.31	0.51					
p_hsch	(F9)	0.21	0.14	0.13	0.28	0.23	0.22	0.31	0.17	0.23				
		0.16	0.14	0.13	0.02	–0.12	0.17	–0.03	0.09	0.11				
		0.23	0.02	–0.02	0.51	0.59	0.23	0.59	0.28	0.34				
p_unemp	(F10)	0.18	0.15	0.14	0.14	0.13	0.18	0.17	0.21	0.19	0.14			
		0.04	0.15	0.13	0.14	0.13	0.06	0.11	0.02	0.06	0.13			
		0.37	0.07	0.12	0.07	–0.01	0.35	0.25	0.43	0.37	0.12			
p_moved	(F11)	0.16	0.13	0.15	0.31	0.19	0.14	0.20	0.20	0.17	0.14	0.13		
		0.11	0.13	0.11	–0.02	0.17	0.13	0.17	0.09	0.10	0.13	0.13		
		0.24	–0.06	–0.18	0.60	0.16	0.07	0.19	0.33	0.27	0.07	0.05		
p_foreign	(F12)	0.14	0.34	0.14	0.19	0.60	0.18	0.40	0.18	0.18	0.17	0.14	0.20	
		0.14	0.18	0.14	0.15	0.11	0.18	0.02	0.10	0.15	0.07	0.14	0.20	
		–0.03	0.40	–0.09	0.19	0.70	0.00	0.61	0.28	–0.17	0.32	–0.01	0.10	

GRDR = gonorrhea diagnosis rate; dens = population density (F1); p_female = proportion female (F2); p_young = proportion aged ≤30 years (F3); p_hisp = proportion Hispanic (F4); p_black = proportion non-Hispanic black (F5); p_xwhite = proportion of minority race/ethnicity (F6); p_single = proportion not currently married (F7); p_pov = proportion below the federal poverty level (F8); p_hsch = proportion with less than a high school education (F7); p_unemp = proportion unemployed (F10); p_moved = proportion moved in the past 12 months (F11); p_foreign = proportion foreign-born (F12).

For example, the correlation between the gonorrhea diagnosis rate and the proportion of CPOA population who are minority race/ethnicity (p_xwhite), or PMC (rate, p_xwhite) = 0.54 and the MIC (rate, p_xwhite) = 0.43. The non-linearity measure for the two variables is 0.14, indicating a somewhat non-linear relationship. On the other hand, when both variables are log transformed, PMC (log_rate, q-xwhite) = 0.65 but MIC (log_rate, q_xwhite) remains the same—0.43. The non-linearity measure between the two log transformed variables has declined to almost zero (0.43–0.65^2^), indicating that the relationship between the log transformed variables is almost linear. 

**Figure 2:**
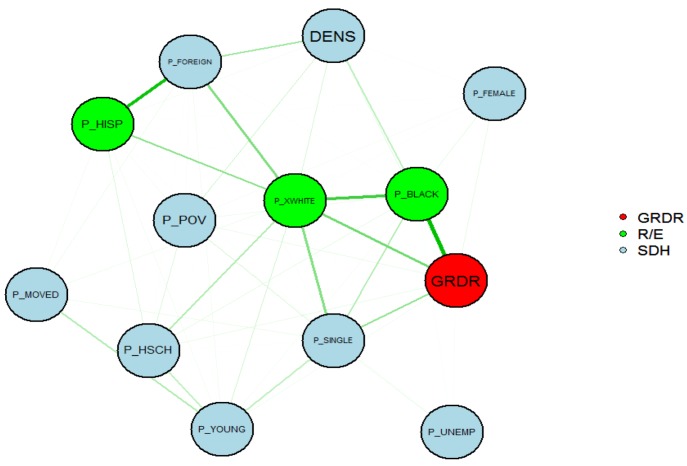
Strength of non-linear associations among both demographic and SDH variables and the gonorrhea diagnosis rate. GRDR represents gonorrhea diagnosis rate and R/E represents the race/ ethnicity groups.

The largest measure for non-linearity is for the relationship between the proportion of the CPOA population who are non-Hispanic black (p_black) and population density (density). The MIC (p_black, density) = MIC (q_black, log-dens) = 0.29, PMC (p_black, density) = 0.13, and PMC (q_black, log_dens) = 0.46, leading to non-linear measures of 0.27 and 0.08 for the original variables and the log transformed variables, respectively. In this case, the log transformation successfully transformed the non-linear relationship to a linear relationship. The MIC and non-linear measures provide a method to verify the effectiveness of transformations used for PMCs. The strengths of the non-linear association between any two variables were presented in Figure 2. The thickness of the line connecting two nodes (variables) represents the relative strength of the association between the two variables. 

The non-linear associations between race ethnicity (R/E) variables and gonorrhea diagnosis rate (GRDR) were stronger than non-linear associations between the SDH variables in the graph. Only the pairs of variables that have an MIC greater than 0.16 were connected by a line.

## 4. Discussion and Conclusions

Our study explored the association between gonorrhea diagnosis rate and each of several SDH and demographic variables. The geographic unit of analysis was the CPOA, a mixed group of county and PUMA (CPOA), as defined in the ACS. Several authors have studied associations between health outcomes and area-based SDH and demographic measures at different geographic levels such as zip-code, census tract, census block, county, and state level. The rationale for studying area-based measures of SDH is that they provide information not captured by individual-level data. For example, community unemployment levels may affect all individuals living within a community, regardless of whether or not they are unemployed [20]. Similarly, the proportion of the CPOA population living below the federal poverty level in a neighborhood may be a marker for neighborhood-level factors potentially related to health [20]. Although the choice of geographic level may affect the analysis results of the area-based measures, Song *et al*. explained the advantages of using the COPA in analyzing the association of AIDS diagnosis rates with SDH variables. The same advantages apply to our analysis of gonorrhea diagnosis rates and SDH variables: (1) CPOA population is large enough to produce stable gonorrhea diagnosis rates; (2) CPOA does not change over time; and (3) CPOA best approximates the geographic scope of regular social interactions. 

We measured the associations between gonorrhea rates and both SDH and demographic variables using correlations and partial correlations. We also calculated the non-linear associations among these variables using MICs and a measure of non-linearity between variables. MICs remained the same, regardless of whether the variables were log transformed. The partial correlation between two variables adjusted for a third variable does not depend on the value at which the third variable is held. However, for non-linear associations (MICs) this may not hold and further methodological research is required to extend the MIC to a measure similar to partial correlation for PMC [15]. One could also use path analysis to examine causal patterns among gonorrhea diagnosis rate and both demographic and SDH variables.

The gonorrhea diagnosis rate is strongly correlated with the proportion of the CPOA population who are non-Hispanic black, proportion who are of minority race/ethnicity, and proportion not currently married. These demographic variables were also strongly correlated with AIDS diagnosis rate [5]. The population density, proportion female, and proportion below the poverty level are moderately correlated with gonorrhea diagnosis rate. However, the partial correlations of these variables vary widely when adjusted for other variables considered in this analysis. The reasons for these variations have been examined in several studies of associations between gonorrhea rate and both SDH and demographic variables conducted at the local level.

An analysis of neighborhood socio-cultural factors influencing the spatial pattern of gonorrhea in North Carolina using cases reported between 2005 and 2008, found that a high percentage of single mothers, more women than men, and low socioeconomic status (SES) appear to influence gonorrhea rates [12]. The neighborhood-level percentage of population who are single mothers and percentage black were strongly correlated, suggesting that neighborhoods with a high proportion of single black mothers experienced a disproportionate burden of infection [12]. In our study, the correlation between proportion not currently married and proportion non-Hispanic black was 0.53; and proportion female is moderately correlated with gonorrhea diagnosis rate. 

Another study of associations between the characteristics of counties and the rates of reported gonorrhea in the southeastern region of the United States from 1986 to 1995 found that when adjusted for variables measuring aspects of social structure, such as a race-based income distribution and residential segregation, the proportion of blacks no longer had an effect on rates of gonorrhea [21]. On the other hand, a study that focused on the longitudinal impact of community characteristics on gonorrhea at the census tract level in New York State showed that increases in gonorrhea rates related to the proportion of the non-Hispanic black population within urban census tracts persisted after multiple SES variables were controlled [22]. Using homicide rates as a proxy for incarceration rates, a study of neighborhood factors affecting STDs in Chicago found that incarceration rates were associated with gonorrhea rates a year later [23]. Even though these studies have shown varying results in different localities, a common theme is the association between gonorrhea rates and both the SDH and demographic variables found to be strongly or moderately associated with gonorrhea diagnosis rates in our analysis.

To conduct an extensive study of relationships of both the SDH and demographic variables to the gonorrhea rates, more geographic area-based estimates of additional variables are required. Whether considering incarceration rates and crime rates or other measures of SDH, expanding our understanding of SDH and integrating measures across the range of social, political, and economic institutions and structures will permit us to have a clearer understanding of how these factors work in our analysis of the geographic distribution of health and disease. The inclusion of data on both SDH and demographic variables into existing surveillance systems would enhance the analysis of these relationships because the diagnosis rates could be stratified by these variables. 

Together with the collection of more SDH variables and methods to measure non-linear associations, geographic area-based analysis of disease incidence and SDH can add value to public health prevention and control programs. If a local or state health department or a community-based organization can pinpoint the local factors affecting gonorrhea rates, then more effective programs can be tailored to the populations they serve. By using science-based evidence to improve these programs, public health officials can hope for greater support of public programs among various stakeholders. Public health professionals may consider collecting such data or encouraging other partners to collect these data that can be used to improve intervention programs. In this way, coordinated, multisectoral efforts may help to address disparities in the distribution of gonorrhea diagnosis rates and help to control the spread of such diseases.

## Appendix

**Figure A1:**
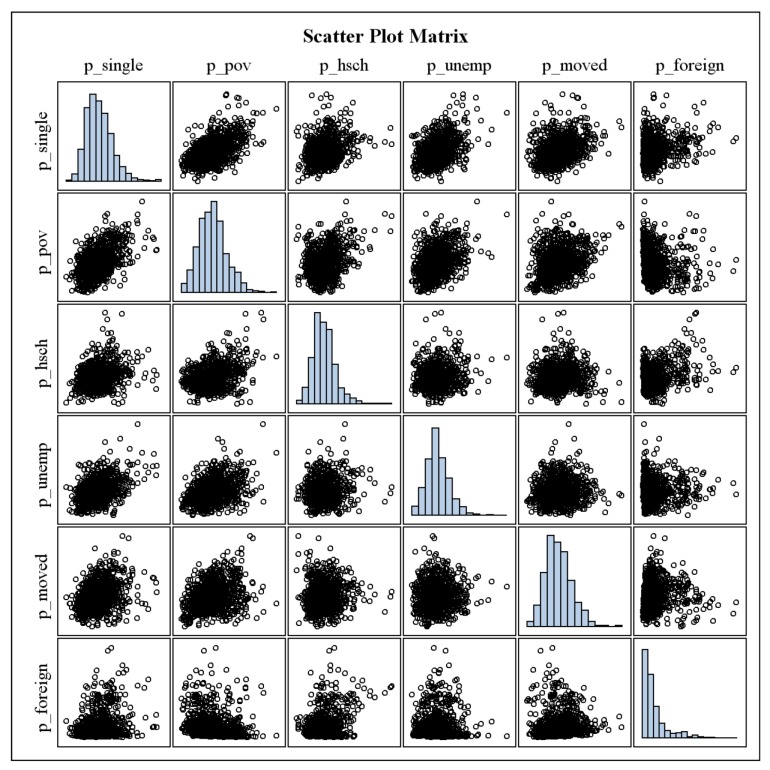
Histograms and scatter plots of the gonorrhea diagnosis rates and both SDH and demographic variables using the original variables (without log transformation): National Notifiable STD Surveillance and American Community Survey data, 2006–2008.

## References

[1] Centers for Disease Control and Prevention Establishing a Holistic Framework to Reduce Inequities in HIV, Viral Hepatitis, STDs, and Tuberculosis in the United States.

[2] Harrison K.M., Dean H.D. (2011). Use of data systems to address social determinants of health: A need to do more. Public Health Rep..

[3] Krieger N., Chen J.T., Ebel G. (1997). Can we monitor socioeconomic inequalities in health? A survey of U.S. health departments’ data collection and reporting practices. Public Health Rep..

[4] Beltran V.M., Harrison K.M., Hall H.I., Dean H.D. (2011). Collection of social determinants of health measures in U.S. national surveillance systems for HIV, viral hepatitis, STDs, and TB. Public Health Rep..

[5] Song R., Hall H.I., Harrison K.M., Sharpe T.T., Lin L.S., Dean H.D. (2011). Identifying the impact of social determinants of health on disease rates using correlation analysis of area-based summary information. Public Health Rep..

[6] Soto K., Petit S., Hadler J.L. (2011). Changing disparities in invasive pneumococcal disease by socioeconomic status and race/ethnitity in Connecticut, 1998–2008. Public Health Rep..

[7] Commer K.F., Grannis S., Dixon B., Bodenhamer D.J., Wiehe S. (2011). Incorporating geospatial capacity within clinical data systems to address social determinants of health. Public Health Rep..

[8] Walkup J., Akincigil A., Hoover D.R., Siegel M.J., Amin S., Crystal S. (2011). Use of medicaid data to explore community characteristics associated with HIV prevalence among beneficiaries with schizophrenia. Public Health Rep..

[9] Centers for Disease Control and Prevention Sexually Transmitted Disease Surveillance 2010.

[10] Hogben M., Leichliter J.S. (2008). Social determinants and sexually transmitted disease disparities. Sex Transm. Dis..

[11] Laumann E.O., Youm Y. (1999). Racial/ethnic group differences in the prevalence of sexually transmitted diseases in the United States: A network explanation. Sex Transm. Dis..

[12] Sullivan A.B., Gesink D.C., Brown P., Zhou L., Fitch M., Serre L., Miller W.C. (2011). Are neighborhood socio-cultural factors influencing the spatial pattern of gonorrhea in North Carolina?. Ann. Epidemiol..

[13] Newman L.M., Berman S.M. (2008). Epidemiology of STD disparities in African American communities. Sex Transm. Dis..

[14] Bolan G.A., Sparling P.F., Wasserheit J.N. (2012). The emerging threat of untreatable gonococcal infection. N. Engl. J. Med..

[15] Speed T. (2011). A correlation for the 21st century. Science.

[16] Reshef D.N., Reshef Y.A., Finucane H.K., Grossman S.R., McVean G., Turnbaugh P.J., Lander E.S., Mitzenmacher M., Sabeti P.C. (2011). Detecting novel associations in large data sets. Science.

[17] MIT News. http://web.mit.edu/newsoffice/2011/large-data-sets-algorithm-1216.html.

[18] MINE: Maximal Information-Based Nonparametric Exploration. http://www.exploredata.net/.

[19] Epskamp S., Cramer A.O.J., Waldorp L.J., Schmittmann V.D., Borsboom D. (2012). Qgraph: Network visualizations of relationships in psychometric data. J. Stat. Softw..

[20] Diez-Roux A.V. (1998). Bringing context back into epidemiology: Variables and fallacies in multilevel analysis. Am. J. Public Health.

[21] Thomas J.C., Garfield M.E. (2003). Social structure, race, and gonorrhea rates in the Southern United States. Ethn. Dis..

[22] Ping D., McNutt L., O’Campo P, Coles F.B. (2009). Changes in community socioeconomic status and racial distribution associated with gonorrhea rates: An analysis at the community level. Sex Transm. Dis..

[23] Thomas J.C., Torrone E.A., Browning C.R. (2010). Neighborhood factors affecting rates of sexually transmitted diseases in Chicago. J. Urban Health.

